# Activation of the α1β2γ2L GABA_A_ Receptor by Physiological Agonists

**DOI:** 10.3390/biom11121864

**Published:** 2021-12-11

**Authors:** Spencer R. Pierce, Allison L. Germann, Gustav Akk

**Affiliations:** Department of Anesthesiology, The Taylor Family Institute for Innovative Psychiatric Research, Washington University School of Medicine, St. Louis, MO 63110, USA; spencerp@wustl.edu (S.R.P.); germanna@wustl.edu (A.L.G.)

**Keywords:** GABA_A_ receptor, activation, potentiation, orthosteric agonist, allosteric agonist

## Abstract

The Cl^−^ permeable GABA_A_ receptor is a major contributor to cellular inhibition in the brain. The receptor is normally activated by synaptically-released or ambient GABA but is sensitive to a number of physiological compounds such as β-alanine, taurine, and neurosteroids that, to various degrees, activate the receptor and modulate responses either to the transmitter or to each other. Here, we describe α1β2γ2L GABA_A_ receptor activation and modulation by combinations of orthosteric and allosteric activators. The overall goal was to gain insight into how changes in the levels of endogenous agonists modulate receptor activity and influence cellular inhibition. Experimental observations and simulations are described in the framework of a cyclic concerted transition model. We also provide general analytical solutions for the analysis of electrophysiological data collected in the presence of combinations of active compounds.

## 1. Introduction

The γ-aminobutyric acid type A (GABA_A_) receptor is expressed in the brain and a number of other organs such as the stomach, kidney and liver, as well as in endocrine tissues and immune cells [1,2,3,4]. The receptor is permeable to Cl^−^. In mature excitable cells, where intracellular [Cl^−^] is low, its activation leads to hyperpolarization of the cell or dampening of the effects of excitatory ion channels. This directly contributes to excitation-inhibition balance in the brain. The GABA_A_ receptor is normally activated by the transmitter GABA, but other endogenous compounds such as β-alanine (3-aminopropanoic acid), taurine (2-aminoethanesulfonic acid), and many neurosteroids can also activate the receptor or modulate activity elicited by the transmitter [5,6,7].

Receptor exposure to these compounds can be phasic (i.e., short-lived) in which receptors in the postsynaptic membrane respond to presynaptically-released GABA. In this scenario, the concentration of the transmitter rises rapidly in the synaptic cleft to low millimolar levels activating the vast majority of receptors, followed by a rapid decline as the transmitter diffuses out of the cleft or is removed by GABA transporters [8,9]. Alternatively, or additionally, the GABA_A_ receptor is tonically exposed to systemically distributed active compounds in the cerebrospinal fluid or those synthesized locally. These include β-alanine, taurine, and numerous neuroactive steroids. Additionally, the low levels of ambient GABA can tonically activate the GABA_A_ receptor, particularly the high-affinity δ subunit-containing receptor that is located outside the synapse [10]. While changes in the ambient levels of GABAergic compounds are commonplace, these are generally slow (at least when compared to the speed of synaptic transmission).

The GABA_A_ receptor is thus simultaneously exposed to multiple active compounds. The net effect of combinations of active compounds on receptor function is predominantly determined by whether the compounds interact with the same or distinct binding sites. In general, combinations of agonists interacting with distinct sites lead to strong potentiation, whereas combinations of agonists that share a binding site are associated with weak potentiation. For example, a neurosteroid that binds to an allosteric site strongly potentiates the response to the orthosteric agonist GABA, whereas taurine or β-alanine that bind to the transmitter binding site are only weak potentiators of currents elicited by GABA [11,12]. The magnitude of response to an agonist combination also depends on the binding and gating properties of each compound.

Here, we describe activation of the α1β2γ2L GABA_A_ receptor in the presence of multiple physiological agonists. The data and simulations are discussed in the framework of a two-state (Resting-Active) cyclic concerted transition model [13,14]. The focus is on physiological agonists of the receptor; additionally, we provide general solutions for analysis of electrophysiological data in the presence of agonist combinations.

## 2. Results and Discussion

We have analyzed and estimated current responses from the α1β2γ2L GABA_A_ receptor in the presence of individual orthosteric and allosteric endogenous agonists and combinations of these compounds. We focused on the transmitter GABA, orthosteric agonists β-alanine and taurine, and allosterically acting neurosteroids allopregnanolone (3α5αP), pregnanolone (3α5βP), and etiocholanolone. The α1β2γ2L receptor is a major synaptic-type GABA_A_ receptor, and the results are therefore relevant to inhibitory synaptic transmission.

The analysis of concentration-response data for individual agonists was carried out using the state function [13,14]:

(1) $$P_{A,\mathrm{peak}}=\frac{1}{1+L^{*}{\left[\frac{1+[\mathrm{agonist}]/K_{R,\mathrm{agonist}}}{1+[\mathrm{agonist}]/(K_{R,\mathrm{agonist}}c_{\mathrm{agonist}})}\right]}^{N_{\mathrm{agonist}}}}$$

where L^*^ expresses the level of background activity, [agonist] is the concentration of agonist, K_R,agonist_ is the equilibrium dissociation constant for the agonist in the resting receptor, and *c*_agonist_ is the ratio of the equilibrium dissociation constant for the steroid in the active receptor to K_R,agonist_. N_agonist_, the number of binding sites, was constrained to two.

**Figure 1:**
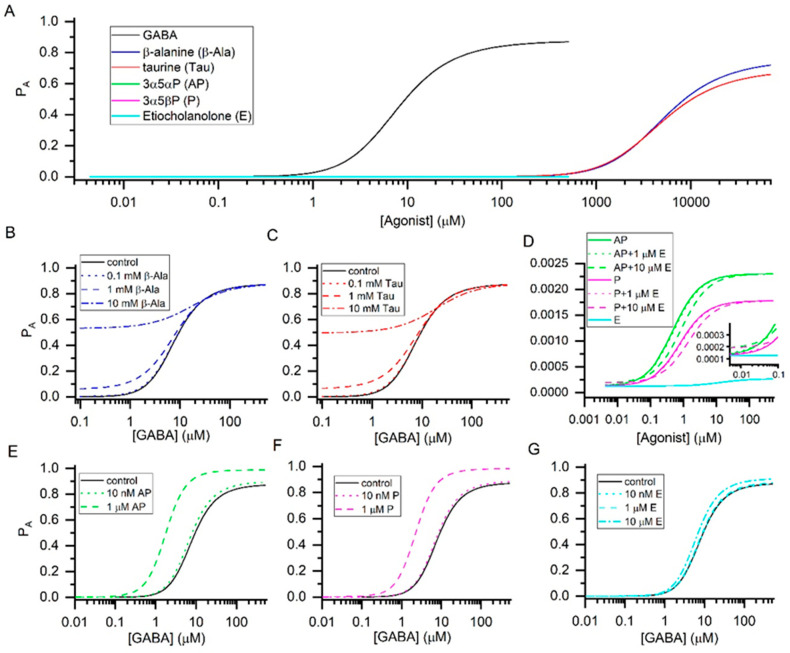
Agonist concentration-response relationships. The graphs show the probability of being in the active state (P_A_) vs. the concentration of agonist. The curves in panel (**A**) were calculated using the K_R_ and *c* values provided in Table 1. The graph indicates that GABA, β-alanine, and taurine are high-efficacy agonists and the neurosteroids 3α5αP, 3α5βP, and etiocholanolone are low-efficacy agonists of the α1β2γ2L receptor. The concentration-response curves for the three steroids are shown at higher resolution in panel (**D**). Panels (**B**,**C**) show modulation of responses to GABA in the presence of 0.1, 1, or 10 mM β-alanine or taurine. While either compound is ineffective at 0.1 mM, potentiation of GABA responses is observed at 1 and 10 mM. Panel D shows activation of the receptor by 3α5αP, 3α5βP, or etiocholanolone, and combinations of 3α5αP or 3α5βP with 1 or 10 µM etiocholanolone. The presence of 10 but not 1 µM etiocholanolone reduces the response to 3α5αP or 3α5βP. Panels (**E**–**G**) show the effects of 3α5αP, 3α5βP, or etiocholanolone on GABA concentration-response curves. Strong potentiation of GABA responses is observed at 1 µM 3α5αP or 3α5βP.

The concentration-response curves in the presence of each agonist individually are given in Figure 1A. The binding and gating properties of the six agonists are presented in Table 1. The data indicate that the orthosteric ligands GABA, β-alanine, and taurine are high-efficacy agonists generating, at saturating concentrations, peak responses with the probability of being in the active state (P_A_) > 0.6. In contrast, the allosteric agonists 3α5αP, 3α5βP, and etiocholanolone are weak, ineffective activators of the α1β2γ2L receptor with maximal P_A_ < 0.003.

Analysis of current responses in the presence of two agonists binding to the same set of sites was carried out using the following equation [11]:

(2) $$P_{A,\mathrm{peak}}=\frac{1}{{1+L}^{*}{\left[\frac{{1+[\mathrm{agonist}1]/K}_{R,\mathrm{agonist}1}{+[\mathrm{agonist}2]/K}_{R,\mathrm{agonist}2}}{{1+[\mathrm{agonist}1]/(K}_{R,\mathrm{agonist}1}c_{\mathrm{agonist}1}{)+[\mathrm{agonist}2]/(K}_{R,\mathrm{agonist}2}c_{\mathrm{agonist}2})}\right]}^{N_{\mathrm{agonist}}}}$$

where agonist1 and agonist2 indicate the paired agonists. Other terms are as described above. The equation can be expanded to include additional agonists by adding repeating terms to the denominator.

In this model, coapplication of two or more agonists is predicted to modulate the current response due to increase in the effective concentration of agonist (i.e., through “concentration additivity”). In the simplest case, the combination of equal concentrations of two agonists with same binding (K_R_) and gating (*c*) properties is functionally equivalent to doubling the concentration of either agonist applied alone. We emphasize that coapplication of a low-efficacy agonist with a high-efficacy agonist is expected to reduce the response to the latter through competitive inhibition.

The endogenous orthosteric agonists GABA, β-alanine, and taurine have relatively similar gating efficacies (Table 1), and are therefore predicted to generate a larger response when applied in combination. Using Equation (2), we have calculated receptor activity in the presence of a range of concentrations of GABA, and selected concentrations (0.1, 1, and 10 mM) of β-alanine or taurine (Figure 1B,C). Both compounds are ineffective at 0.1 mM but potentiate responses to GABA at 1 and 10 mM. The basal physiological concentrations of β-alanine and taurine are <0.1 mM [16,17] indicating that the native α1β2γ2L receptor is not activated or modulated, to a meaningful extent, by endogenous β-alanine or taurine. Ionophoretically or bath-applied β-alanine and taurine reduce the excitability of spinal neurons, and evoke outward current in thalamic relay neurons [18,19]. At least for taurine, the effects take place at physiologically relevant concentrations (50 µM). We infer that other subtypes of the GABA_A_ receptor, possibly the extrasynaptic α4β2δ receptor [19,20], underlie the physiological GABAergic actions of taurine.

Combinations of allosteric agonists interacting with the same site(s) behave similarly to combinations of orthosteric agonists. Combination of agonists with similar efficacies leads to an increase in response due to concentration additivity, while the application of a low-efficacy agonist decreases the response to a higher-efficacy agonist. Inspection of the properties of neurosteroids in Table 1 indicates that a combination of 3α5αP and 3α5βP increases the response to either steroid alone, since the steroids have similar gating efficacies (values of *c*). In contrast, coapplication of etiocholanolone decreases the response to 3α5αP or 3α5βP through competitive inhibition. The findings are summarized in Figure 1D.

Analysis of receptor activity in the presence of two agonists binding to distinct sites was carried out using the following equation [21,22]:

(3) $$P_{A,\mathrm{peak}}=\frac{1}{1+L^{*}{\left[\frac{1+[\mathrm{agonist}1]/K_{R,\mathrm{agonist}1}}{1+[\mathrm{agonist}1]/(K_{R,\mathrm{agonist}1}c_{\mathrm{agonist}1})}\right]}^{N_{\mathrm{agonist}1}}{\left[\frac{1+[\mathrm{agonist}2]/K_{R,\mathrm{agonist}2}}{1+[\mathrm{agonist}2]/(K_{R,\mathrm{agonist}2}c_{\mathrm{agonist}2})}\right]}^{N_{\mathrm{agonist}2}}}$$

All terms have been described above. The equation can be expanded to include additional agonists by adding terms to the denominator. In this model, combined agonists modulate receptor function through “energetic additivity”. At low agonist concentrations, the combined effect is highly synergistic (i.e., the response to the combination is greater than the sum of responses to each agonist administered alone).

We modeled responses to GABA in the presence of 3α5αP (10 nM and 1 µM), 3α5βP (10 nM and 1 µM), or etiocholanolone (10 nM, 1 µM, and 10 µM). At 10 nM all steroids were relatively ineffective, whereas 1 µM 3α5αP and 3α5βP shifted the GABA concentration-response relationship to the left and enhanced the P_A_ at saturating GABA. Etiocholanolone was ineffective also at 1 µM, due to its lower affinity to the receptor, but weakly potentiated the receptor at 10 µM. The findings are summarized in Figure 1E–G.

In terms of GABA_A_ receptor function, the steroids 3α5αP and 3α5βP are mutually interchangeable. In practical terms, a combination of physiologically relevant concentrations [23] of 10 nM 3α5αP and 10 nM 3α5βP activates the receptor nearly equivalently to 20 nM of either steroid applied alone. The GABA_A_ receptor in the brain is exposed to additional species of potentiating steroids (e.g., tetrahydrodeoxycorticosterone, allotetrahydrodeoxycorticosterone and others), likely with affinities and efficacies similar to those of 3α5αP [24,25], the combination of which may elevate the net concentration of potentiating neurosteroids to one producing a meaningful effect on GABA_A_ receptor function. We also note that during pregnancy circulating neurosteroids likely reach the levels capable of modulating GABAergic activity [26].

The presence of a low-efficacy agonist competitively inhibits activity. For example, etiocholanolone, that is a weak agonist of the α1β2γ2L receptor, inhibits the response to 3α5αP (or 3α5βP) but potentiates the response to GABA through energetic additivity. In the combination of GABA, 3α5αP and etiocholanolone, the latter enhances receptor function at low concentrations of 3α5αP but inhibits at high concentrations of 3α5αP. These considerations are also relevant in the case of GABAergic clinical drugs. While etiocholanolone is an anticonvulsant [27], its administration, particularly at high doses, can reduce the physiological GABAergic tone imposed by high-efficacy steroids (3α5αP, 3α5βP).

## 3. Concluding Remarks

We have shown here that the numerous agonists to which the native GABA_A_ receptor is exposed under physiological conditions act through concentration additivity or energetic additivity to modulate receptor function. In energetic additivity, an agonist binding to a distinct site strongly potentiates the control response. For example, neurosteroids are efficacious potentiators of responses elicited by GABA. In concentration additivity, agonists interacting with the same sites lead to a relatively modest increase in response, as long as the agonists have similar gating efficacies. This scenario applies to the combinations of GABA, β-alanine and taurine, or the combination of neurosteroids 3α5αP and 3α5βP. 

## Figures and Tables

**Table 1:** Binding and gating properties of some physiological agonists of the GABA_A_ receptor. The table gives the equilibrium dissociation constants in the resting receptor (K_R_), the ratios of equilibrium dissociation constants in active and resting receptors (*c*), and maximal P_A_ for selected orthosteric (GABA, β-alanine, taurine) and allosteric agonists (3α5αP, 3α5βP, etiocholanolone) in the α1β2γ2L receptor. The data for GABA and taurine are from [15], and the data for the three steroids from [11]. The values of K_R_ and *c* for β-alanine were estimated from fits of concentration-response data (n = 5 cells) to Equation (1), with the value of L constrained to 8000 and the number of binding sites (N) held at 2. The experiments were conducted and data analyzed as described previously [11]. Maximal P_A_ is calculated as 1/(1 + L × *c*^N^).

Agonist	K_R_ (µM)	*c*	Maximal P_A_
GABA	16 ± 3	0.0042 ± 0.0003	0.88 ± 0.06
β-Alanine	6554 ± 1026	0.0064 ± 0.0010	0.75 ± 0.12
Taurine	5100 ± 1200	0.0075 ± 0.0006	0.69 ± 0.06
3α5αP	0.27 ± 0.07	0.233 ± 0.018	0.0023 ± 0.0002
3α5βP	0.45 ± 0.06	0.265 ± 0.010	0.0018 ± 0.0001
Etiocholanolone	11.1 ± 1.5	0.685 ± 0.009	0.0003 ± 0.0001
